# Development and internal-external validation of a risk prediction model for acute pain after HAIC for patients with liver cancer using logistic regression and XGBoost algorithm

**DOI:** 10.1016/j.apjon.2026.100923

**Published:** 2026-02-18

**Authors:** Jiacheng Cao, Yina Gong, Fan Wang, Jiayang Zhang, Chunyan Chen, Jiawei Cao, Minghui Xie, Wenjuan Zhao

**Affiliations:** a Department of Nursing, Fudan University Shanghai Cancer Center/ Department of Oncology, Shanghai Medical College, Fudan University, Shanghai, China; b Department of Nursing, Zhongshan Hospital Affiliated to Fudan University, Shanghai, China; c Department of Interventional Therapy, Changhai Hospital, Naval Medical University, Shanghai, China

**Keywords:** Acute pain, HAIC, Prediction model, Risk classification, XGBoost, Logistic regression

## Abstract

**Objective:**

To develop a clinical model for the early prediction of moderate-to-severe pain after Hepatic Artery Infusion Chemotherapy (HAIC) in liver cancer patients using XGBoost algorithm and then compare its prediction capacity with the logistic model.

**Methods:**

A multicenter cohort study presented according to TRIPOD + AI statement, which was conducted in 3 tertiary hospitals in Shanghai from May 2022 to June 2024. Lasso regression was used to screen for risk factors. Logistic regression and XGBoost algorithm were tested and compared by Brier, area under the curve (AUC), calibration curve, Hosmer–Lemeshow test, intercept and slope, and decision curve analysis (DCA).

**Results:**

The study included 1303 patients, with 725 for model development, 578 for external validation. In the XGBoost model, the top 3 most important variables were oxaliplatin dosage, initial HAIC treatment and age. XGBoost model and logistic regression model showed discriminative ability with AUC values of 0.729, 0.714, 0.707 and 0.722, 0.715, 0.684 in the modeling, internal validation, and external validation sets, respectively. The calibration and decision curve analyses of both models showed favorable results in both modeling and validation sets, except for the calibration of logistic regression model in external validation. XGBoost model performed better across all evaluated dimensions in external validation. Based on the risk score generated by the XGBoost model, the population was categorized into low, intermediate, and high-risk subgroups for stratification.

**Conclusions:**

XGBoost model has higher accuracy and stronger robustness in predicting acute moderate-to-severe pain after HAIC in patients with liver cancer, which will facilitate risk assessment and implement precise and early interventions.

## Introduction

Liver cancer is one of the most common malignant tumors globally, ranking sixth in incidence and third in mortality.^1^ Radical surgery is considered the optimal treatment for liver cancer.^2^ However, many patients are ineligible for surgery and are thus restricted to non-surgical treatments or conversion therapy for potentially resectable liver cancer.^2^^,^^3^ Hepatic Artery Infusion Chemotherapy (HAIC) has been widely used in the treatment of unresectable liver cancer due to its effectiveness and minimal loss of normal liver tissue.^4^^,^^5^ Over 40% of patients undergoing HAIC experience acute postoperative pain, with approximately 25% reporting it as moderate or severe.^6^^,^^7^ Acute postoperative pain may hinder early postoperative recovery, prolong hospital stays, and even compromise patient adherence to treatment regimens.^8^, ^9^, ^10^

Accurate preoperative identification of postoperative pain risk is critical for patients' early pain management.^11^ In this regard, predicting acute postoperative pain occurring after treatments like hepatectomy or TACE has become a matter of research interest in recent years.^12^^,^^13^ However, few studies have focused on predicting acute postoperative pain following HAIC, with an absence of adequate external validation to support clinical application.^14^ Furthermore, the inclusion of predictors that are not easily assessed in practice limits the model's practical clinical utility. Therefore, more tailored and optimized models with clinical utility are required for liver cancer patients undergoing HAIC who share a particular profile. Acute postoperative pain in these patients stems from procedure-related injury, arterial chemotherapy infusion, and individual factors.^15^

Due to the research gap mentioned above, this study aimed to develop and internally-externally validate a clinical prediction model with robustness and clinical usability for the early prediction of moderate-to-severe pain after HAIC in liver cancer patients based on multi-center datasets. The optimal model was determined by comparing the performance between the logistic algorithm and the XGBoost algorithm.

## Methods

### Design

This study employed a mixed cohort design, utilizing one cohort for the model development phase and another cohort for the model validation phase. The study was reported in line with the TRIPOD + AI (Transparent Reporting of a Multivariable Prediction Model for Individual Prognosis or Diagnosis, Extended for Artificial Intelligence) guidelines.^16^

### Data source and patient population

Adult patients hospitalized in three tertiary hospitals in Shanghai for HAIC were identified. For HAIC procedure, the Seldinger technique was employed to perform femoral artery catheterization. A catheter was advanced to the tumor-feeding artery under imaging guidance. Following catheter placement, patients were returned to their ward to receive continuous arterial infusion of chemotherapy drugs while maintained at bed rest. The chemotherapy regimens administered via HAIC included FOLFOX (5-fluorouracil, leucovorin and oxaliplatin), GEMOX (gemcitabine and oxaliplatin), oxaliplatin plus raltitrexed, GP (gemcitabine and cisplatin) and irinotecan plus 5-fluorouracil. The exclusion criteria were presented as follows: 1) use of psychotropic drugs; 2) use of sedative drugs; 3) cognitive impairment; 4) concurrent hepatectomy combined with HAIC; 5) concurrent hepatic artery embolization combined with HAIC; and 6) incomplete records of included variables > 30%. The data for model development were collected from patients from May 2022 to December 2023 in a tertiary hospital. The data for external validation was collected from patients during January 2024 to June 2024 in three tertiary hospitals. All data were collected from the electronic medical record system of hospitals, starting from May 5, 2024, when the research protocol had been ethically approved. No patient recruitment or prospective interaction occurred prior to the ethics approval date.

### Outcomes

The outcome of the prediction model was moderate-to-severe postoperative acute pain, assessed using the Numeric Rating Scale (NRS-11). The NRS-11 is a self-reported scale of pain intensity with a rating of 0–10. Scores are typically categorized as follows: 0 indicates no pain, 1–3 indicates mild pain, 4–6 indicates moderate pain, and 7–10 indicates severe pain.^17^ NRS-11 was reported by patients and collected by well-trained nurses to the study. Nurses assessed pain once on return to the ward after surgery, hourly after starting HAIC, and every two hours after completion of HAIC until discharge in the following day, and recorded it in the electronic medical record system. Given that postoperative pain refers to acute pain occurring within seven days following procedure,^18^ pain scores from the immediate postoperative period until seven days post-procedure (including in-hospital and follow-up assessments) were included. Pain assessment of discharged patients was followed up by telephone and recorded by a follow up specialist, still using NRS-11. The highest pain score was selected as a determination of whether moderate-to-severe pain had occurred postoperatively. Moderate-to-severe pain was defined as NRS-11 ≥ 4.

### Predictors

Based on the symptom experience model,^19^ through a literature scoping review and an expert meeting in **Supplementary File 1** and **2**, 17 candidate predictors were initially considered, including demographic characteristics, disease characteristics and individual characteristics. Demographic characteristics included age and sex. Disease characteristics included disease diagnosis, C-reactive protein (mg/L), HAIC regimen, oxaliplatin dosage (mg), history of hepatectomy, history of TACE, ECOG score, history of hypertension, history of diabetes mellitus, history of chronic hepatitis, and history of gastroduodenal ulcers. Individual characteristics included times of HAIC treatment, history of previous postoperative pain after HAIC, history of pre-operative chronic cancer pain and history of alcohol use. Among these candidate predictors, age, sex, history of TACE, times of HAIC treatment and history of previous postoperative pain after HAIC may be associated with individual's sense of pain; ECOG score, history of hypertension and history of diabetes may be associated with physiological state; disease diagnosis, history of hepatectomy, history of gastroduodenal ulcers and history of alcohol use may be associated with tissue damage at the site of administration; C-reactive protein (mg/L), history of chronic hepatitis and history of pre-operative chronic cancer pain may be associated with inflammatory reaction. All predictors were measured one day before HAIC.

### Sample size

#### Model development

In this study, sample size was calculated using the clinical prediction model sample size calculation formula EPV (Events Per Variable), shown in Eq. (1).^20^

(Eq. 1) $$N=10\times \frac{k}{p}$$

In this formula, k represents the number of independent variables and p represents the minimum proportion of negative or positive cases in the overall population. According to the relevant literature, the incidence of moderate-to-severe post-operative acute pain in adults with liver cancer undergoing HAIC was estimated 25%.^7^ This resulted in a final sample size of 680 cases.

#### External validation

In this study, the calculation of the external validation sample size was performed using the experiential guidelines for external validation sample size for clinical prediction models. According to the experiential guidelines, the external validation set needed to contain at least 100 samples with an outcome event and 100 samples without an outcome event.^21^ Based on the literature review, the incidence of moderate-to-severe post-operative acute pain in adults with liver cancer undergoing HAIC was estimated as 25%,^7^ which was calculated to result in an external validation sample size of at least 400 cases.

### Data cleaning and quality checks

We performed data quality checks during data cleaning, including assessment of potential duplicate entries, outliers and the extent of missing data. Samples missing outcomes were excluded. Multiple imputation was applied to impute missing values of candidate predictors. The same characteristic determination methods were applied to all centers.

### Statistical analysis

#### Cohort and outcome description

A database was set up using EXCEL for data entry and collation; data were statistically analyzed using SPSS (version 20.0, IBM, American) and R (version 4.3.3; R Core Team, Vienna, Austria). Missing data were addressed using multiple imputation implemented via “mice” package of R. All variables were included in the imputation models. Descriptive statistics were used to summarize the distribution of all the candidate predictors and the outcome. Normally distributed and non-normally distributed variables were shown as mean ± standard deviation and median (interquartile range), respectively. Categorical variables were presented as numbers (percentage). *Independent Student's t* test and the *Mann–Whitney U test* were respectively performed to analyze the difference between 2 groups of normally distributed and non-normally distributed variables. The $\chi^{2}$ *test* or the *Fisher test* was conducted to compare the difference of categorical variables. A 2-sided *P* value of < 0.05 was considered to be of statistical significance.

#### Model development

The modeling set was used for model development. All continuous variables were not converted to categorical variables. *Least absolute shrinkage and selection operator (LASSO)* was used for predictor selection. Two model types were developed and compared: a logistic regression model (implemented using the “rms” package in R) and an XGBoost model (implemented using the “xgboost” package in R). These two models were subsequently trained and tested in the modeling set and external validation set, respectively. A threshold of 0.5 was used for model performance evaluation. The Brier score was calculated for overall performance. The closer the Brier score was to 0, the better the overall model performance was.^22^ Area under the curve (AUC) was calculated for discrimination. AUC > 0.7 indicates acceptable model differentiation.^23^ Calibration curve, *Hosmer–Lemeshow test*, intercept and slope were calculated for calibration. The calibration curve fit the target line, the *Hosmer–Lemeshow test P* value > 0.05, the intercept was close to 0 and the slope was close to 1 indicated that the model was well calibrated.^22^ Decision curve analysis (DCA) was calculated for clinical benefit.

#### Internal validation

Internal validation was performed by using the mean estimate of 1000 bootstrapping, with 95% confidence intervals (95% CI) used to assess the robustness of each performance metric across the resampling. Brier score was calculated for overall performance. AUC was calculated for discrimination. *Hosmer–Lemeshow test*, intercept and slope were calculated for calibration.

#### External validation

The data for external validation from three centers were used in external validation. The predictive performance was evaluated both collectively and sequentially across the three datasets. Brier score was calculated for overall performance. AUC was calculated for discrimination. *Hosmer–Lemeshow test*, intercept and slope were calculated for calibration. Decision curve analysis (DCA) was calculated for clinical benefit. Subgroup analyses based on sex were also carried out to assess fairness in model performance by *Delong test*.

#### Risk stratification system construction

Risk stratification of predicted values was performed based on the 95% sensitivity and 95% specificity values of the Receiver Operating Characteristic (ROC) curves of the modeling set of the optimal prediction model.

## Results

### Study population characteristics

**Fig. 1:**
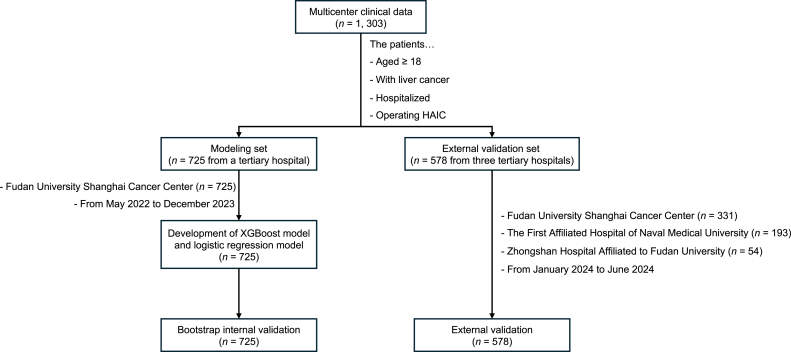
Flowchart of study population.

**Fig. 2:**
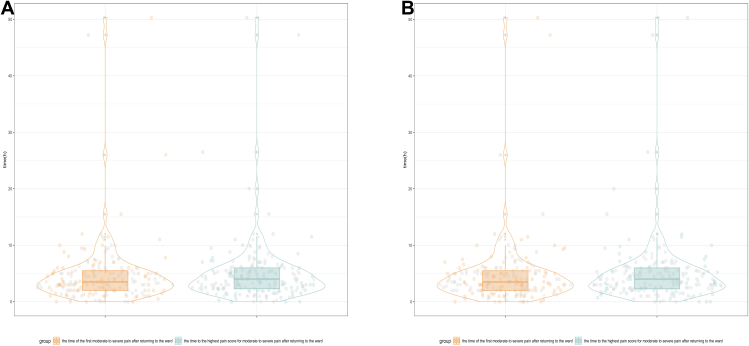
(A) Violin plot of the occurrence time of pain of modeling set; (B) Violin plot of the occurrence time of pain of testing set.

Flowchart of study population is shown in Fig. 1. In the modeling set, a total of 725 samples were collected. In the external validation set, a total of 578 samples were collected. Details on missing values are provided in **Supplementary File 3**. Baseline characteristics of the study population are summarized in Table 1, which demonstrates significant differences between the modeling set and testing set in demographic characteristics, disease characteristics and individual characteristics. Moderate-to-severe postoperative acute pain occurred in 184/725 (25.4%) and 102/578 (17.6%) patients in the modeling and validation sets, respectively. The distribution of pain occurrence time in both modeling set and external validation set is shown in Fig. 2.

**Table 1:** Study population characteristics of modeling set, testing set and all patients [*N* (%)].

Characteristics	All patients (*n* = 1303)	Modeling set (*n* = 725)	Testing set (*n* = 578)	*P* value
**Age (years) [M (P_25_, P_75_)]**	57.0 (49.0, 65.0)	56.0 (47.0, 65.0)	58.5 (52.0, 65.0)	< 0.001^a^
**Sex**				0.014^a^
Male	1062 (81.5)	608 (83.9)	454 (78.5)	
Female	241 (18.5)	117 (16.1)	124 (21.5)	
**Initial HAIC treatment**				0.037^a^
Yes	448 (34.4)	267 (36.8)	181 (31.3)	
No	855 (65.6)	458 (63.2)	397 (68.7)	
**History of previous postoperative pain after HAIC**				< 0.001^a^
Yes	136 (10.4)	110 (15.2)	26 (4.5)	
No	1167 (89.6)	615 (84.8)	552 (95.5)	
**History of pre-operative chronic cancer pain**				**0.556**
Yes	52 (4.0)	31 (4.3)	21 (3.6)	
No	1251 (96.0)	694 (95.7)	557 (96.4)	
**History of alcohol use**				**0.596**
Yes	213 (16.4)	115 (15.9)	98 (17.0)	
No	1090 (83.6)	610 (84.1)	480 (83.0)	
**Primary liver cancer**				< 0.001^a^
Yes	991 (76.1)	605 (83.4)	386 (66.8)	
No	312 (23.9)	120 (16.6)	192 (33.2)	
C-reactive protein (mg/L) [M (P_25_, P_75_)]	8.1 (2.6, 27.1)	8.3 (2.8,28.3)	8.0 (2.2,26.1)	0.345
**HAIC regimen**				< 0.001^a^
Including oxaliplatin	1167 (89.6)	672 (92.7)	495 (85.6)	
Excluding oxaliplatin	136 (10.4)	53 (7.3)	83 (14.4)	
Oxaliplatin dosage (mg) [M (P_25_, P_75_)]	125.0 (100.0, 150.0)	130.0 (100.0, 150.0)	100.0 (100.0, 150.0)	0.001^a^
**History of hepatectomy**				**< 0.001**^a^
Yes	495 (38.0)	329 (45.4)	166 (28.7)	
No	808 (62.0)	396 (54.6)	412 (71.3)	
**History of TACE**				< 0.001^a^
Yes	498 (38.2)	228 (31.4)	270 (46.7)	
No	805 (61.8)	497 (68.6)	308 (53.3)	
**ECOG score**				**0.372**
0	1 (0.1)	1 (0.1)	0 (0.0)	
1	1302 (99.9)	724 (99.9)	578 (100.0)	
**History of diabetes mellitus**				**0.039**^a^
Yes	202 (15.5)	99 (13.7)	103 (17.8)	
No	1101 (84.5)	626 (86.3)	475 (82.2)	
**History of hypertension**				**< 0.001**^a^
Yes	447 (34.3)	210 (29.0)	237 (41.0)	
No	856 (65.7)	515 (71.0)	341 (59.0)	
**History of chronic hepatitis**				< 0.001^a^
Yes	678 (52.0)	437 (60.3)	241 (41.7)	
No	625 (48.0)	288 (39.7)	337 (58.3)	
**History of gastroduodenal ulcers**				0.473
Yes	17 (1.3)	8 (1.1)	9 (1.6)	
No	1286 (98.7)	717 (98.9)	569 (98.4)	

HAIC, hepatic artery infusion chemotherapy; TACE, transcatheter arterial chemoembolization; ECOG, Eastern Cooperative Oncology Group. M (P25, P75), Median (25th percentile, 75th percentile).

a *P* < 0.05.

### Model development

**Fig. 3:**
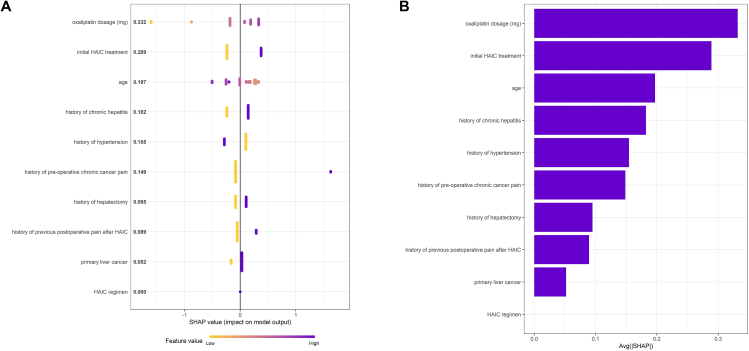
(A) Beeswarm plot of SHAP of XGBoost model; (B) Bar plot of SHAP of XGBoost model.

**Fig. 4:**
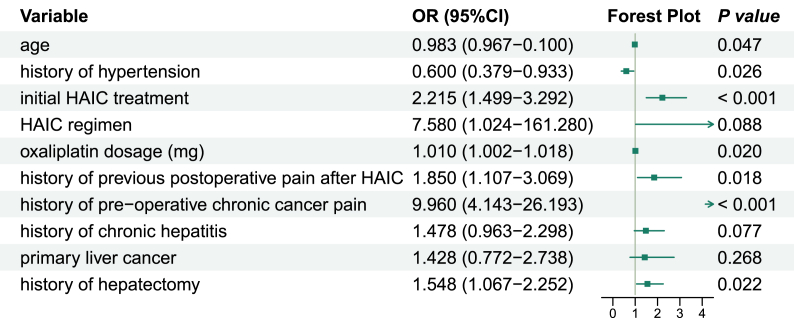
Forest plot for logistic regression model.

**Fig. 5:**
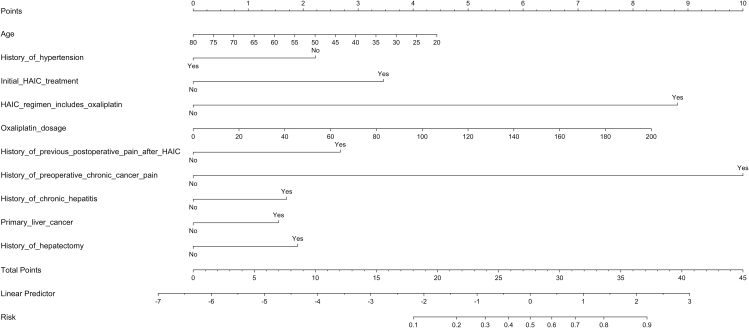
Nomogram for logistic regression model.

Seventeen candidate predictors were screened and 10 predictors were finally included in the model via LASSO regression. **Supplementary File 4** shows the coefficient profile plot of the variable coefficient paths for a fitted LASSO. The importance plot of variables of XGBoost model is shown in Fig. 3 as SHAP values, predictors’ detection ability was ordered from high to low: oxaliplatin dosage, initial HAIC treatment, age, history of chronic hepatitis, history of hypertension, history of pre-operative chronic cancer pain, history of hepatectomy, history of previous postoperative pain after HAIC, primary liver cancer and HAIC regimen. These predictors were subsequently incorporated into multivariable logistic regression (Fig. 4, Fig. 5), and the VIF of predictors confirmed absence of multicollinearity (**Supplementary File 5**). Algorithm-specific parameters and hyperparameters are detailed in **Supplementary File 6.**

### Prediction capacity comparison in two models

In terms of overall performance, the Brier score of the XGBoost model showed 0.163, 0.166 (95% CI: 0.164–0.169) and 0.134, while the logistic regression model showed 0.164, 0.166 (95% CI: 0.164–0.169) and 0.136 in the respective sets.

**Fig. 6:**
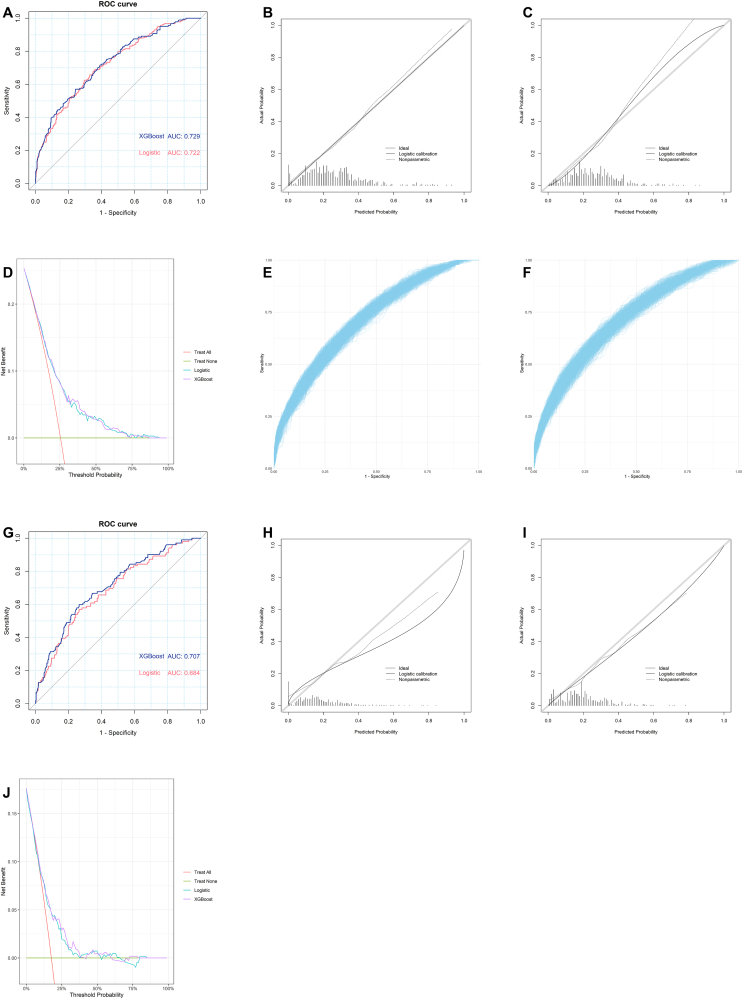
Models' performances. ROC curve in modeling set; (B) Calibration curve of logistic regression model in modeling set; (C) Calibration curve of XGBoost model in modeling set; (D) DCA in modeling set; (E) ROC curve of logistic regression model in internal validation; (F) ROC curve of XGBoost model in internal validation; (G) ROC curve in testing set; (H) Calibration curve of logistic regression model in testing set; (I) Calibration curve of XGBoost model in testing set; (J) DCA in testing set. ROC, receiver operating characteristic curve; DCA, decision curve analysis.

In terms of discriminative ability, the AUC of the XGBoost model showed 0.729 (95% CI: 0.687–0.772), 0.714 (95% CI: 0.698–0.727) and 0.707 (95% CI: 0.652–0.762) in the modeling, internal validation, and external validation sets, while the logistic regression model showed values of 0.722 (95% CI: 0.680–0.765), 0.715 (95% CI: 0.703–0.722) and 0.684 (95% CI: 0.626–0.741) in the respective sets (Fig. 6).

In terms of calibration, the *P* value of the Hosmer–Lemeshow test of the XGBoost model showed 0.351, 0.256 (95% CI: 0.003–0.840) and 0.244 in the modeling, internal validation, and external validation sets, while the logistic regression model showed values of 0.821, 0.196 (95% CI: 0.001–0.816) and < 0.001 in the respective sets. For the intercept, the XGBoost model showed 0.217, 0.140 (95% CI: −0.136-0.498) and −0.252, while the logistic regression model showed < 0.001, −0.161 (95% CI: −0.419-0.151) and −0.571 in the respective sets. For the slope, the XGBoost model showed 1.297, 1.155 (95% CI: 0.911–1.436) and 0.919, while the logistic regression model showed 1.000, 0.825 (95% CI: 0.587–1.107) and 0.575 in the respective sets. The calibration curves are shown in Fig. 6.

In terms of clinical benefit, the DCA of the XGBoost model was above the two reference lines in the range of 5%–85% in the modeling sets, and 5%–8%, 42%–60% and 73%–79% in the external validation sets, while the logistic regression model showed range of 5%–93%, and 10%–64% and 80%–84% in the respective sets (Fig. 6).

We evaluated the model's performance across different sexes. In the validation set, XGBoost model showed the AUC of 0.720 (95% CI: 0.659–0.781) for males and 0.656 (95% CI: 0.534–0.780) for females, while logistic model showed the AUC of 0.694 (95% CI: 0.631–0.757) for males and 0.642 (95% CI: 0.509–0.694) for females. Furthermore, the *P* value of Delong test for XGBoost model was 0.367 (D = −0.904, df = 187.77), and 0.491 (D = −0.690, df = 181.62) for logistic model, which indicated that there were no differences between patients across different sexes for both models.

### Optimal prediction model deployment

**Fig. 7:**
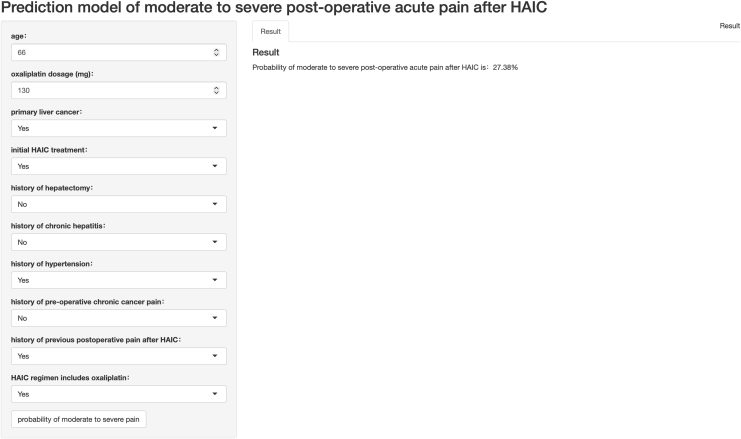
Website of the ShinyAPP.

Since the XGBoost model had better overall performance, discrimination, calibration and clinical benefit in external validation, we finally chose it as the optimal prediction model. The deployment of the model was constructed by ShinyAPP, which could enable users with or without coding skills to use the model as an online calculator (https://jonathancao.shinyapps.io/xgboostshinyeng/), and the screenshot of the website is shown in Fig. 7. In addition, if unavailable data exists, users have to impute it.

### Risk stratification programme construction

**Fig. 8:**
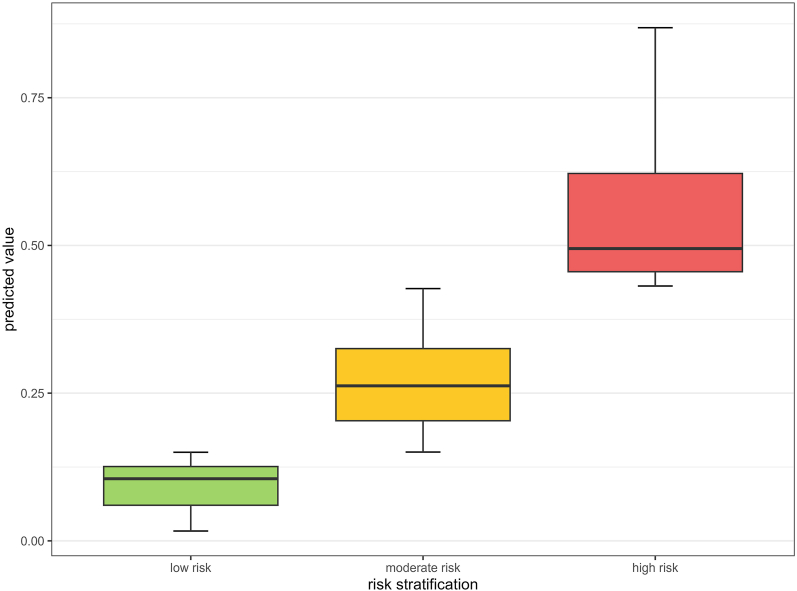
Risk stratification of the modeling set.

Predicted values between 0.15 and 0.43 were recognized as moderate risk and predicted values over 0.43 were recognized as high risk. The risk stratification of the modeling set is shown in Fig. 8.

## Discussion

### Main findings

In this study, we developed and validated an XGBoost-based risk stratification system for predicting postoperative moderate-to-severe pain following HAIC among patients with liver cancer. A comparative analysis demonstrated that the XGBoost model consistently outperformed the logistic regression baseline across all key metrics, achieving a higher AUC (0.707 vs. 0.684), better calibration (intercept: −0.252 vs. −0.571), and a lower Brier score (0.134 vs. 0.136). These results indicate that the XGBoost approach offers more accurate and reliable risk prediction for acute pain after HAIC.

Designed for use prior to the HAIC procedure, the model incorporates 10 routinely available preoperative variables. It demonstrated strong discrimination, calibration, and clinical utility. In external validation, the model achieved a Brier score of 0.134 (close to 0), reflecting excellent overall performance. Its AUC of 0.707 (95% CI: 0.652–0.762) exceeded the conventional threshold of 0.70 for clinical usefulness. Furthermore, the calibration slope remained near 1 (slope = 0.919), supporting both good discriminative ability and reliable probability estimation. These results indicate that the model performs adequately to support its potential integration into clinical practice for risk stratification. Decision curve analysis confirmed its net benefit for patients. Moreover, the use of the ShinyApp and the risk stratification system display the model in a clinically intuitive format, promoting its implementation in routine practice.

Our prediction model addresses a critical clinical need about accurately identifying patients with different risk levels for moderate-to-severe pain following HAIC. Although existing models, such as the model by Li et al.^14^ demonstrated strong discriminative performance, its clinical utility remains limited. For instance, some predictors in the model (e.g., tumor diameter, and tumor-to-capsule distance) are not routinely assessed in clinical practice. Furthermore, the lack of external validation limits the generalizability.

Thus, our study collected multicenter data to validate the robustness of our model. Our modeling set was only derived from one center, and our external validation set was derived from three centers which had considerable baseline heterogeneity of baseline comparing with the modeling set. In this case, our model still shows similar performance in external validation as it does in the modeling set. This result strongly indicates that our model is generalizable and robust, which can be applied to different populations and achieve similarly good performance.^24^

By following the symptom experiences model^19^ and experts’ opinion when considering candidate predictors, our study confirms and expands the knowledge about predictive factors for postoperative moderate-to-severe pain after HAIC among patients with liver cancer, which provides clinicians with a comprehensive risk assessment tool. Symptoms experience model was proposed by Terri S Armstrong^19^ in 2003, categorizing the antecedents of symptoms into demographic characteristics, disease characteristics, and individual characteristics. 10 predictors were finally chosen, including age, first time of HAIC, HAIC regimen, oxaliplatin dose, history of pain after HAIC, chronic cancer pain, chronic hepatitis, primary liver cancer, history of hepatectomy and history of hypertension. Li et al.^14^ chose age, tumor diameter, distance from liver capsule, portal vein tumor thrombus, oxaliplatin preparation time and oxaliplatin manufacturer as predictive factors of their model. Compared with recent studies, despite the absence of imaging factors, our study mainly discovered additional disease-related factors of postoperative moderate-to-severe pain after HAIC among patients with liver cancer. Primary liver cancer is mainly caused by chronic inflammation.^25^ In addition, chronic hepatitis and liver surgery both damage hepatocytes, which can also cause inflammation^25^, ^26^, ^27^. Since inflammation is known to cause pain, these predictive factors can be explained.^28^ Patients with a history of hypertension routinely receive antihypertensive medications, which have the effect of dilating peripheral vascularization.^29^ Since postoperative pain after HAIC is often caused by vascular smooth muscle spasm due to chemotherapeutic drug infusion, and the vasodilator effect of vasodilator antihypertensive drugs can play a role in preventing or alleviating vascular spasm and improving ischemia in tissues, thus preventing patients from experiencing pain due to vasospasm.^30^^,^^31^ Our study further confirms the use and dose of oxaliplatin as predictive factors of postoperative acute pain after HAIC. Previous studies have reported that patients may experience abdominal pain after hepatic arterial infusion of oxaliplatin, and pain relief after slowing down or stopping the infusion.^32^ Therefore, the results of our study are consistent with previous reports. However, the pathophysiological mechanisms of this phenomenon remain unelucidated and warrant further investigation.

Considering that the modeling set is mainly composed of males, we further evaluated the performance of the model across different sexes. Although predictive accuracy was lower in females than in males, the DeLong test of AUC revealed no statistically significant differences.

### Implications for nursing practice and research

In light of the above challenges and the measures, the main strength of this study lies in the clinical value and robustness of the model. Besides, the three-stage risk **s**tratification system with the ShinyAPP enables personalized, precise, and user-friendly management of moderate-to-severe postoperative pain following HAIC. Postoperative pain management is one of the highlights of perioperative care for HAIC.^9^ Prior studies have been conducted to reduce the incidence and intensity of postoperative pain in postoperative HAIC patients by hepatic artery injection of lidocaine or by multimodal analgesia, which resulted in a reduction of morphine use but the precise patterns of pain management remain undefined.^33^^,^^34^ However, these studies employed the same prior pain intervention for every patient, which may lead to medication misuse and waste of health care resources. Current expert consensus emphasizes personalized preventive analgesia prior to surgery.^9^ Our findings establish a basis for stratified pain management patterns for postoperative pain after HAIC. Based on our findings, risk levels of pain can be classified by preoperative assessment prior to HAIC. Furthermore, implementing targeted, individualized preventive measures based on risk levels prior to HAIC procedures can increase the effectiveness of pain management, reduce adverse reactions of analgesic drugs, and avoid waste of medical resources. In future studies, patients undergoing HAIC may be classified into low-risk, moderate-risk and high-risk groups by three-stage risk stratification system from our study to adopt different pain management patterns. For example, for low-risk patients (predicted probability < 0.15), we can provide basic preventions; for moderate-risk patients (0.15 ≤ predicted probability < 0.43), we can provide basic prevention plus non-medicine preventions; for high-risk patients (predicted probability ≥ 0.43), we can provide basic prevention, non-medicine preventions, and medicine-based preventions. In patients identified as moderate-to-high risk, the pain management plan should incorporate both psychological assessment and more frequent pain evaluation.^9^

### Limitations

However, some limitations must be acknowledged. Firstly, although the review of the literature and expert consultation were used to guide the selection of variables based on the symptom experience model, the retrospective design inherently limited inclusion of potentially relevant variables (e.g., imaging data, psychological status, genetic susceptibility, and inflammatory biomarkers). Future models should incorporate these factors to improve predictive accuracy. Secondly, although this study was multicenter, all participating institutions were located within a single geographic region, which may limit the generalizability of the results to other provinces or countries. Further validation in other regions or countries would be valuable before broader implementation. Thirdly, the absence of comparison against additional machine learning algorithms limits the strength of the comparative evaluation. Fourthly, although the model demonstrated acceptable discriminative ability, its predictive performance still remains to be improved. Prediction models using more advanced machine learning algorithms with multicenter population in different regions and more types of candidate predictors should be designed in the future.

## Conclusions

This study developed and validated logistic and XGBoost models for prediction of postoperative moderate-to-severe pain after HAIC, and XGBoost model demonstrated superior performance and robustness. Based on predicted values from XGBoost model, a risk stratification programme was constructed. This programme facilitates clinical assessment of the risk of moderate-to-severe pain after HAIC in liver cancer patients and enables precise and early intervention.

## CRediT authorship contribution statement

**Jiacheng Cao**: Conceptualization, Methodology, Software, Data curation, Formal analysis, Visualization, Writing - Original Draft, Writing - Review & Editing. **Yina Gong:** Conceptualization, Methodology, Validation, Investigation, Resources, Writing - Original Draft. **Jiayang Zhang**: Conceptualization, Writing - Original Draft, Writing - Review & Editing. **Fan Wang:** Conceptualization, Validation, Investigation, Data Curation. **Chunyan Chen:** Investigation, Data Curation. **Jiawei Cao:** Investigation, Data Curation. **Minghui Xie:** Conceptualization, Methodology, Resources, Investigation, Data Curation, Supervision, Writing - Review & Editing, **Wenjuan Zhao:** Conceptualization, Methodology, Resources, Writing - Original Draft, Writing - Review & Editing, Supervision, Project administration, Funding acquisition. All authors have read and approved the final manuscript.

## Ethics statement

This study was approved by the Ethics Board of Fudan University Shanghai Cancer Center (Approval No. 2404294-Exp17, May 5, 2024) and was conducted in accordance with the 1964 Helsinki Declaration and its later amendments or comparable ethical standards. All participants provided written informed consent.

## Data availability statement

The data supporting the findings of this study are available upon reasonable request from the corresponding author. The data are not publicly available due to privacy or ethical restrictions.

## Declaration of generative AI and AI-assisted technologies in the writing process

No AI tools/services were used during the preparation of this work.

## Funding

The study was funded by Huhang Nursing Research Fund of Shanghai Anticancer Association (Grant No. SACA-HH202307) and Fudan University-Fosun Nursing Research Fund (Grant No. FNF202426).

## Declaration of competing interest

The authors declare no conflict of interest.
