# Supplementary material for: Development and internal-external validation of a risk prediction model for acute pain after HAIC for patients with liver cancer using logistic regression and XGBoost algorithm
Source: Asia Pac J Oncol Nurs. 2026 Feb 18;13:100923. doi: 10.1016/j.apjon.2026.100923 (PMC12995816; doi:10.1016/j.apjon.2026.100923)
Supplement: Multimedia component 1 [file mmc1.docx]

**Development and internal-external validation of a risk prediction model for acute pain after HAIC for patients with liver cancer using Logistic Regression and XGBoost Algorithm**

**Supplementary File 1**

**Results of candidate predictors through a literature scoping review**

Through a literature scoping review aimed at factors associated with pain following interventional therapy for liver cancer, variables in Table A.1 were considered as candidate predictors.

**Table A.1.** Candidate predictors through a literature scoping review

| Characteristics | Candidate predictors |
| --- | --- |
| Demographic characteristics | Age^1–13^, sex^3,14^ and BMI^15^. |
| Disease Characteristics | (1) Tumor-related conditions: tumor blood supply^16^, number of tumors in the liver^6,10–12,17–21^, tumor diameter^6,9,10,13,19,21–23^, tumor volume^24^, tumor capsule^25^, distance from tumor to hepatic capsule^1,2,4,12,13,21,26,27^, vascular invasion^19,28,21^, PVTT^6,13,29^, liver cancer stage^7^, hepatic artery diameter^30^ and Child-Pugh score^15^.  Physical condition-related score: ECOG performance status^2,4,11,12,22,31^.  (2) Laboratory indices: prothrombin activity^1,9^, AFP^9^, C-reactive protein^32^, hemoglobin^33^, IL-6^33^, IL-10^33^, TNF-α^33^, MTT^33^, NLR^6^ and PLR^6^. The therapeutic administration protocols of HAIC and TACE are inconsistent. For TACE, associated factors include Adriamycin dose^9^, Pirarubicin mixed with iodized oil for embolization^2,7^, iodized oil dose^1,27,34^, usage of gelatin sponge^4,5,26^, DEB-TACE^7,18–21,28,35–37^, particle size of drug-eluting beads^38^, anhydrous alcohol embolism^27^ and oxaliplatin dosage^39–41^. For HAIC, associated factors include oxaliplatin preparation time and the manufacturer of oxaliplatin^13,30^.  The surgical procedures: for TACE, associated factors include iodide sedimentation type^25^, range of embolization^22,25,35^ and superselective chemoembolization^29^; for HAIC, associated factor includes extrahepatic perfusion^42^.  Medical histories: the number of TACE procedures^11,18–22,24^, liver cancer surgery history^3^, liver cancer transplantation history^3^, drinking history^15^, number of HAIC procedures^14^ and chronic cancer pain^14^.  Complications: ascites^31^, diabetes history^3,15^, chronic liver disease history^8,9,43^ and gastroduodenal ulcer^42^. |
| Individual characteristics | A history of abdominal pain after TACE^18–21,28^, preoperative analgesic administration^43^, intraoperative analgesic administration^43^, pain before surgery^31^, anxiety^5,12,24,33^ and depression^5,33^. |

**References**

1. Yang H, Zhai H, Li H, Liang S, Liu J, Ma H. Construction and verification of a nomogram model for predicting pain after transcatheter arterial chemoembolization in patients with hepatocellular carcinoma. *Chin J Pract Nursi*. 2022;38(24):1885-1891. doi:10.3760/cma.j.cn211501-20211008-02774

2. Luo J, Shao G, Zheng J, et al. The regularity of abdominal pain and its influence factors in patients with The regularity of abdominal pain and its influence factors in patients with. *J Interv Radiol*. 2017;26(7):613-617. doi:10.3969/j.issn.1008-794X.2017.07.009

3. Sun J, Zhou G, Zhang Y, et al. Comprehensive analysis of common safety profiles and their predictive factors in 520 records of liver cancer patients treated by drug-eluting beads transarterial chemoembolization. *Med Baltim*. 2018;97(26):e11131. doi:10.1097/md.0000000000011131

4. Li A, Yang S. Logistic analysis affecting post-TACE pain in patients with primary liver cancer. *Guide China Med*. 2019;17(32):36-37.

5. Tao N, Liang S, Xia H. Analysis on pain-related factors after interventional treatment for middle-advanced primary liver cancer. *J Guangxi Med Univ*. 2022;39(11):1833-1839. doi:10.16190/j.cnki.45-1211/r.2022.11.023

6. Liang Q, Wu D, Wang S, Yan K, Deng L, Han S. Prediction of pain intensity after transarterial chemoembolization in patients with hepatocellular carcinoma by neutrophil to lymphocyte ratio-platelet to lymphocyte ratio score. *Electron J Liver Tumor*. 2022;9(3):27-35. doi:10.3969/j.issn.2095-7815.2022.03.006

7. Song PW, Liu YH, Wang T, Yu L, Liu JL. Construction of pain prediction model for patients undergoing hepatic arterial chemoembolization. *World J Surg Oncol*. 2023;21(1):100. doi:10.1186/s12957-023-02986-y

8. Pachev A, Raynaud L, Paulatto L, et al. Predictive factors of severe abdominal pain during and after transarterial chemoembolization for hepatocellular carcinoma. *Eur Radiol*. 2021;31(5):3267-3275. doi:10.1007/s00330-020-07404-5

9. Benzakoun J, Ronot M, Lagadec M, et al. Risks factors for severe pain after selective liver transarterial chemoembolization. *Liver Int*. 2017;37(4):583-591. doi:10.1111/liv.13235

10. Qin H, Xiao X, Ya H, et al. Nomogram for predicting severe abdominal pain after initial conventional transarterial chemoembolisation for hepatocellular carcinoma: a retrospective study. *Sci Rep*. 2023;13:22397. doi:10.1038/s41598-023-49509-z

11. Yan J. Patterns and influencing factors of post-TACE abdominal pain in patients with primary liver cancer. *Tibet Med J*. 2024;45(4):46-48.

12. Lian Z, Li S, Wang M, Ma X. Occurrence and influencing factors of abdominal pain after TACE for hepatocellular carcinoma. *J Med Forum*. 2025;46(3):260-263, 268. doi:10.20159/j.cnki.jmf.2025.03.008

13. Li J, Shi C, Chen J, Zhao Y, Du J, Song J. Risk Factors and Predictive Analysis of Acute Severe Abdominal Pain After Hepatic Artery Infusion Chemotherapy in Patients with Hepatocellular Carcinoma. *J Hepatocell Carcinoma*. 2025;12:289-299. doi:10.2147/JHC.S494668

14. Zhao W. The pattern and influencing factors of abdominal pain after HAIC in patients with liver cancer. In: Shanghai Nursing Association; 2022:361-362. doi:10.26914/c.cnkihy.2022.029495

15. Gu H. Analysis of factors affecting the degree of postoperative pain in patients with hepatitis B-associated hepatocellular carcinoma treated with TACE. *Health Everyone*. 2016;(14).

16. Li R, Liu X, Lu C. Complications after interventional therapy for primary liver cancer. *J Pract Med*. 2010;26(01):163.

17. Bray F, Laversanne M, Sung H, et al. Global cancer statistics 2022: GLOBOCAN estimates of incidence and mortality worldwide for 36 cancers in 185 countries. *CA Cancer J Clin*. 2024;74(3):229-263. doi:10.3322/caac.21834

18. Zhao Y, Wang K, Liu Z, Jia Y, Qin C. Risk factors for moderate and severe acute abdominal pain in patients with HCC after TACE and predictive model establishment. *Anhui Med Pharm J*. 2023;27(2):366-369. doi:10.3969/j.issn.1009-6469.2023.02.035

19. Cai P, Tian W, Wang Yi, An X, Tian L, Wang M. Construction of a predictive model for acute abdominal pain after transcatheter arterial chemoembolization for hepatocellular carcinoma. *Mod Interv Diagn Treat Gastroenterol*. 2021;26(11):1400-1403. doi:10.3969/j.issn.1672-2159.2021.11.013

20. Bian L, Gao B, Zhang S, et al. Risk factors and predictive analysis of acute severe abdominal pain after transarterial chemoembolization. *Chin J Nurs*. 2020;55(3):416-421. doi:10.3761/j.issn.0254-1769.2020.03.020

21. Tian C, Chen A, Du X, et al. Prediction Model of Moderate and Severe Abdominal Pain after TACE for Hepatocellular Carcinoma Based on CT Imaging and Clinical Multivariate Regression Analysis. *Chin J CT MRI*. 2024;22(5):109-112.

22. Yang Y, Chen S, Yan Z, Jiao Y, Yan X, Li Y. Construction and Validation of Prediction Model of Severe Abdominal Pain Post-Transarterial Chemoembolization in Patients with HBV-Associated Primary Liver Cancer. *Comput Math Methods Med*. 2022;2022:5203166. doi:10.1155/2022/5203166

23. You K, Wang J, Xu J, et al. Development and validation of a predictive scoring system for post-transarterial chemoembolization pain management in liver cancer patients. *J Gastrointest Oncol*. 2024;15(1):425-434. doi:10.21037/jgo-24-2

24. Zhou B, Chen M, Chen Q, Chen Y, Peng B. Relationship between Preoperative Psychological State and Acute Postoperative Pain after TACE for Hepatocellular carcinoma. *Jilin Med J*. 2016;37(6):1500-1501,1502. doi:10.3969/j.issn.1004-0412.2016.06.125

25. Shen H, Yang G, Liu R, Liu Y, Yang Y, Yue T. The clinical significance of liver pain in patients with primary hepatocellular carcinoma after transcatheter arterial chemoembolization. *J Interv Radiol*. 2010;19(4):297-300. doi:10.3969/j.issn.1008-794X.2010.04.009

26. Meng Y, Chen H, Zhai X, Chen Z. Influencing Factors on the Postoperative Pain of Transcatheter Arterial Chemoembolization in Patients with Primary Liver Cancer. *J Chin Oncol*. 2013;19(09):722-725.

27. Bao J, Cui C, Lin Z, et al. Development and validation of predictive model for moderate to severe abdominal pain after first-time transarterial chemoembolization in patients with hepatocellularcarcinoma. *J Nanjing Med Univ Sci*. 2024;44(5):655-660.

28. Bian LF, Zhao XH, Gao BL, et al. Predictive model for acute abdominal pain after transarterial chemoembolization for liver cancer. *World J Gastroenterol*. 2020;26(30):4442-4452. doi:10.3748/wjg.v26.i30.4442

29. Wang TC, Zhang ZS, Xiao YD. Determination of Risk Factors for Pain After Transarterial Chemoembolization with Drug-Eluting Beads for Hepatocellular Carcinoma. *J Pain Res*. 2020;13:649-656. doi:10.2147/jpr.S246197

30. Wu Z, Guo W, Chen S, Zhuang W. Determinants of pain in advanced HCC patients recieving hepatic artery infusion chemotherapy. *Invest New Drugs*. 2021;39(2):394-399. doi:10.1007/s10637-020-01009-x

31. Hinrichs JB, Hasdemir DB, Nordlohne M, et al. Health-Related Quality of Life in Patients with Hepatocellular Carcinoma Treated with Initial Transarterial Chemoembolization. *Cardiovasc Intervent Radiol*. 2017;40(10):1559-1566. doi:10.1007/s00270-017-1681-6

32. Hartrumpf KJ, Marquardt S, Werncke T, et al. Quality of life in patients undergoing repetitive TACE for the treatment of intermediate stage HCC. *J Cancer Res Clin Oncol*. 2018;144(10):1991-1999. doi:10.1007/s00432-018-2704-7

33. Zhang J, Wang H, Ye R. Observation on the influencing factors of pain after interventional intervention for liver cancer and the effect of nursing plan. *J Logist Univ PAPMedical Sci*. 2021;30(8).

34. Li D. *Adverse Reactions and Its Influencing Factors in the Therapy of Primary Liver Cancer after Transcatheter Arterial Chemoembolization*. master. Bengbu Medical University; 2015.

35. Du Q. *Abdominal Pain after Transarterial Chemoembolization in Patients with Hepatocellular Carcinoma: Incidence and Predictors*. master. Anhui Medical University; 2023.

36. Du QQ, Liang M, Jiang B, et al. Incidence and predictors of abdominal pain after transarterial chemoembolization of hepatocellular carcinoma: a single-center retrospective study. *Eur J Oncol Nurs*. 2023;66:102355. doi:10.1016/j.ejon.2023.102355

37. Song PW, Wang JL, Wang T, Zou HN, Liu YH. Creating and Testing a Model to Predict Postoperative Discomfort in Patients with Hepatocellular Carcinoma Receiving Transarterial Chemoembolisation. *Hepat Mon*. 2023;23(1). doi:10.5812/hepatmon-133918

38. Chen D, Xiao S, Rao Y, Liu J. Observation and nursing care for adverse reactions of drug-eluting beads transcatheter arterial chemoembolization for the treatment of primary hepatocellular carcinoma. *J Nurs Sci*. 2020;35(05):38-40.

39. Li R, Wu L, Liu X. Study on the correlation between different doses of oxaliplatin by TACE treatment and liver function, immune function indexes, and adverse reactions in patients with primary hepatocellular carcinoma. *J China Prescr Drug*. 2022;20(3):84-86.

40. Yang J, Liu J, Yang J, Jiang X, Jiang X, Wang W. Elderly patients with hepatocellular carcinoma treated with transcatheter arterial chemoembolization with low doses of oxaliplatin. *Chin J Hepatobiliary Surg*. 2015;21(6):401-404. doi:10.3760/cma.j.issn.1007-8118.2015.06.011

41. Li G, Yu X, Xie P, Pu H. Study on the Doses of Oxaliplatin in Patients with Hepatocellular Carcinoma after Transcatheter Arterial Chemoembolization. *China Pharm*. 2016;27(18):2470-2472.

42. Kerbage F, Grinda T, Smolenschi C, et al. Is intra-arterial hepatic chemotherapy painful? *Support Care Cancer*. 2020;28(10):4585-4587. doi:10.1007/s00520-020-05560-4

43. Cai X, Li H, Qiu J, et al. Construction and verification of a pain risk prediction model for patients with liver cancer after TACE. *Chin J Clin Ed*. 2024;18(8):722-728. doi:10.3877/cma.j.issn.1674-0785.2024.08.004

**Supplementary File 2**

**Results of candidate predictors through an expert meeting**

**Expert inclusion criteria**

Invite a total of five medical and nursing experts engaged in liver cancer interventional therapy or cancer pain research to participate in the meeting. The inclusion criteria for the experts were: (1) Bachelor's degree or above; (2) intermediate title or above; (3) ≥ 5 years of clinical work or management in medicine or nursing in liver cancer interventional therapy or cancer pain research.

**General information about the expert**

According to the expert inclusion criteria, a total of five medical and nursing experts in liver cancer interventional therapy or cancer pain participated in the expert meeting. Please see Table A.2 for the general information of experts.

**Table A.2.** General information on correspondence experts (n=5)

| Item | n | (%) |
| --- | --- | --- |
| Age |  |  |
| 30-39 | 2 | 40.00 |
| 40-49 | 2 | 40.00 |
| 50-59 | 1 | 20.00 |
| Career |  |  |
| Doctor | 3 | 60.00 |
| Nurse | 2 | 40.00 |
| Working area |  |  |
| Interventional therapy | 3 | 60.00 |
| Combination of Chinese and Western medicine | 2 | 40.00 |
| Degree |  |  |
| Bachelor | 1 | 20.00 |
| Master | 2 | 40.00 |
| Doctor | 2 | 40.00 |
| Title |  |  |
| Intermediate title | 2 | 40.00 |
| Deputy senior professional title | 3 | 60.00 |
| Working years |  |  |
| 11-15 | 3 | 60.00 |
| 16-20 | 1 | 20.00 |
| 21-25 | 1 | 20.00 |

**Expert authority coefficient**

The familiarity of the 5 experts with the research is shown in Table A.3, and the familiarity coefficient (Cs) was calculated to be 0.96. The results of expert judgment basis are shown in Table A.4, and the judgment coefficient (Ca) was calculated to be 0.98. According to the calculation formula of expert authority coefficient(Cr), the Cr of experts was calculated to be 0.97＞0.8. It indicated that the experts consulted in this study had high authority and the results are reliable.

**Table A.3.** Frequency distribution table of expert familiarity

| Category | Familiar | | More familiar | | General | | Not familiar | | Unfamiliar | |
| --- | --- | --- | --- | --- | --- | --- | --- | --- | --- | --- |
|  | n | % | n | % | n | % | n | % | n | % |
| Statistics | 4 | 80.0 | 1 | 20.0 | 0 | 0.0 | 0 | 0.0 | 0 | 0.0 |

**Table A.4.** Frequency distribution table of expert judgment degree

| Judgments based | Great impact | | Moderate impact | | Little impact | |
| --- | --- | --- | --- | --- | --- | --- |
|  | n | % | n | % | n | % |
| Theoretic analysis | 4 | 80.0 | 1 | 20.0 | 0 | 0.0 |
| Practice or research experience | 5 | 100.0 | 0 | 0.0 | 0 | 0.0 |
| Relevant information | 4 | 80.0 | 1 | 20.0 | 0 | 0.0 |
| Intuitive feeling | 1 | 20.0 | 2 | 40.0 | 2 | 40.0 |

**Results**

Through the expert meeting, experts modified candidate predictors from literature review and added a few candidate predictors by clinical experience and accessibility. 17 candidate predictors were finally determined. The following comments were given by the expert meeting:

(1) Suggested deletions: hepatic artery diameter, distance from tumor to hepatic envelope, vascular invasion, stage of liver cancer, portal vein cancer thrombus, number of intrahepatic tumor lesions, presence of extrahepatic perfusion, abdominal swelling, plasminogen activity, AFP, hemoglobin, interleukin-6, interleukin-8, TNF-alpha, NLR, PLR, SAS score, SDS score, Child-Pugh, history of diabetes, and oxaliplatin manufacturer.

(2) Suggested deletion of surgical modality-type influences present in TACE but not in HAIC.

(3) Suggested additions: history of hypertension, primary liver cancer.

(4) It was recommended that the phrase “preoperative oral pain medication” be changed to “chronic cancer pain”.

(5) Based on clinical experience and literature reports, it was suggested that HAIC regimen be included as a predictor in the prediction model.

**Supplementary File 3**

**Data cleaning of modeling set and external validation set**

Duplicate entries were checked manually. Outliers were checked by Gaussian Mixture Model (GMM) and manual checking. Both duplicate entries and outliers were not found in modeling set and external validation set. The extent of missing data is shown in Fig A.1.





**Fig A.1.** (A) The extent of missing data in modeling set; (B) The extent of missing data in external validation set

**Supplementary File 4**

**Results of LASSO**

The LASSO coefficient paths and cross validation error are shown in Figure A.2. Table A.5 shows the variables’ coefficient of LASSO regression. In the condition of lambda.1se = 0.016, 10 variables were finally selected as candidate predictors.

**

**

**Fig A.2.** (A) LASSO coefficient paths plot; (B) Cross validation error plot

**Table A.5.** Variables’ coefficient of LASSO regression

| Variable | Lambda.min | Lambda.1se |
| --- | --- | --- |
| Age | -0.015 | -0.011 |
| Sex | - | - |
| Primary liver cancer | 0.298 | 0.119 |
| Initial HAIC treatment | 0.714 | 0.468 |
| History of hepatectomy | 0.368 | 0.155 |
| History of TACE | -0.052 | - |
| History of chronic hepatitis | 0.353 | 0.267 |
| ECOG score | 0.817 | - |
| History of diabetes mellitus | -0.078 | - |
| History of hypertension | -0.424 | -0.277 |
| History of gastroduodenal ulcers | -0.440 | - |
| History of alcohol use | 0.061 | - |
| C-reactive protein (mg/L) | -0.002 | - |
| History of pre-operative chronic cancer pain | 2.174 | 1.575 |
| History of previous postoperative pain after HAIC | 0.541 | 0.280 |
| Oxaliplatin dosage (mg) | 0.008 | 0.008 |
| HAIC regimen | 1.470 | 0.433 |

**Supplementary File 5**

**VIF of predictors**

| Predictors | VIF |
| --- | --- |
| Age | 1.135 |
| History of hypertension | 1.131 |
| Initial HAIC treatment | 1.174 |
| HAIC regimen | 1.310 |
| Oxaliplatin dosage | 1.303 |
| History of previous postoperative pain after HAIC | 1.167 |
| History of preoperative chronic cancer pain | 1.074 |
| History of chronic hepatitis | 1.257 |
| Primary liver cancer | 1.257 |
| History of hepatectomy | 1.074 |

**Supplementary File 6**

**Parameters or hyper****parameters of two** **algorithms**

The parameter or hyperparameter of two algorithms were shown in Table A.6. Considering of the balance of discrimination and calibration, we tuned the hyperparameters of XGBoost model by manual. Besides, the hyperparameter “subsample” of XGBoost model was used to handle the class imbalance.

**Table A.6.** The parameter or hyperparameter of logistic regression model and XGBoost model

| Algorithm | Parameter or hyperparameter |
| --- | --- |
| Logistic regresion | Logit（P）= -4.471-0.017×age-0.511×history of hypertension+0.795×initial HAIC treatment+2.026×HAIC regimen+0.010×oxaliplatin dosage+0.615×history of previous postoperative pain after HAIC+2.299×pre-operative chronic cancer pain+0.391×history of chronic hepatitis+ 0.357×primary liver cancer+ 0.437×history of hepatectomy |
| XGBoost | set.seed (121), max_depth=1, nthread=32, objective="binary:logistic", subsample=0.6, eval_metric="auc", eta=0.15, scale_pos_weight=1.01, nrounds=70 |
